# Optimal doses of sevoflurane and propofol in rabbits

**DOI:** 10.1186/1756-0500-7-820

**Published:** 2014-11-19

**Authors:** Yoshihide Terada, Tadahiko Ishiyama, Nobumasa Asano, Masakazu Kotoda, Kodai Ikemoto, Noriyuki Shintani, Daniel I Sessler, Takashi Matsukawa

**Affiliations:** Department of Anesthesiology, Faculty of Medicine, University of Yamanashi, 1110 Shimokato, Chuo, Yamanashi, 409-3898 Japan; Surgical Center, University of Yamanashi Hospital, University of Yamanashi, 1110 Shimokato, Chuo, Yamanashi, 409-3898 Japan; Department of Outcomes Research, The Cleveland Clinic, Cleveland, Ohio USA

**Keywords:** Bispectral index, Propofol, Sevoflurane, Rabbits, Anesthesia

## Abstract

**Background:**

Although sevoflurane and propofol are commonly used anesthetics in rabbits, optimal doses of remain unclear. We thus assessed the optimal hypnotic doses of sevoflurane and propofol, and evaluated the influence of dexmedetomidine on sevoflurane and propofol requirements.

**Methods:**

Twenty-eight Japanese white rabbits were randomly assigned to one of four groups (n = 7 each). Rabbits were given either sevoflurane, propofol, sevoflurane + dexmedetomidine, or propofol + dexmedetomidine (injected 30 μg∙kg^-1^∙hr^-1^ for 10 min followed by an infusion of 3.5 μg∙kg^-1^∙hr^-1^). Hypnotic level was evaluated with Bispectral Index (BIS), a well-validated electroenchalographic measure, with values between 40 and 60 representing optimal hypnosis. BIS measurements were made 10 minutes after the adjustment of target end-tidal sevoflurane concentration in the sevoflurane group and sevoflurane + dexmedetomidine group, and at 10 min after the change of infusion rate in the propofol group and propofol + dexmedetomidine group.

**Results:**

BIS values were linearly related to sevoflurane concentration and propofol infusion rate, with or without dexmedetomidine. Sevoflurane concentration at BIS = 50 was 3.9 ± 0.2% in the sevoflurane group and 2.6 ± 0.3% in the sevoflurane + dexmedetomidine group. The propofol infusion rate to make BIS = 50 was 102 ± 5 mg∙kg^-1^∙hr^-1^ in the propofol group, and 90 ± 10 mg∙kg^-1^∙hr^-1^ in the propofol + dexmedetomidine group.

**Conclusions:**

The optimal end-tidal concentration of sevoflurane alone was thus 3.9%, and optimal infusion rate for propofol alone was 102 mg∙kg^-1^∙hr^-1^. Dexmedetomidine reduced sevoflurane requirement by 33% and propofol requirement by 11%.

## Background

Anesthesia is required for many animal experiments, and often provided by sevoflurane or propofol. Those agents are also used in anesthesia for rabbits [1]. Nevertheless, optimal anesthetic doses of sevoflurane and propofol in rabbits remain unclear. Dexmedetomidine, a central alpha-2 receptor agonist, is sedative and also has hypotensive, analgesic, and anesthetic sparing properties [2]. The extent to which dexmedetomidine reduces the need for sevoflurane and propofol in rabbits remains unknown.

The best-established monitor of hypnotic (anesthetic) depth is the Bispectral Index (BIS) which is based on human electroencephalogram, but has also been used in animals [3, 4]. In this study we sought to clarify the BIS monitoring usefulness in establishing the optimal doses of sevoflurane or propofol in rabbits and also its usefulness in evaluating the influence of dexmedetomidine on sevoflurane and propofol anesthetic requirements in rabbits. Therefore, our primary goal was to determine the relationship between BIS and sevoflurane concentration or propofol dose in rabbits. Our secondary goal was to determine the optimal hypnotic doses of sevoflurane and propofol, and the extent to which dexmedetomidine reduced anesthetic requirement.

## Methods

Experiments were performed on 28 healthy Japanese white rabbits weighing 2.8-3.9 kg. The Committee on Animal Research, University of Yamanashi, Yamanashi, Japan, approved this study. The acclimation period of the rabbits was more than 3 weeks. The rabbits were randomly assigned to one of four groups (sevoflurane, propofol, sevoflurane and dexmedetomidine, and propofol and dexmedetomidine; n = 7 in each group) based on anesthetic methods. After obtaining intravenous access in an ear vein, each rabbit was given either sevoflurane 5% in oxygen via mask or propofol 10 mg∙kg^-1^ iv followed by an infusion of 50 mg∙kg^-1^∙hr^-1^. Rocuronium 5 mg∙kg^-1^ was injected simultaneously. Rocuronium was injected intermittently as needed. Then the animals were tracheostomized under local anesthesia with 1% lidocaine and their lungs were mechanically ventilated with an oxygen-air mixture. End-tidal CO_2_ was continuously monitored (Vamos, Dräger medical, Tokyo, Japan), and the tidal volume and respiratory rate were adjusted to maintain arterial carbon dioxide tension between 35 and 45 mmHg. A femoral artery catheter was inserted to monitor arterial blood pressure. Bicarbonated Ringer’s solution was infused at 10 mL∙kg^-1^∙h^-1^. After completion of tracheotomy, sevoflurane was decreased to 1% in the sevoflurane anesthetized groups, and dexmedetomidine was injected 30 μg∙kg^-1^∙hr^-1^ for 10 min (5 μg∙kg^-1^) followed by an infusion of 3.5 μg∙kg^-1^∙hr^-1^ in the sevoflurane and dexmedetomidine group. In the propofol group, infusion rate of propofol was continued at 50 mg∙kg^-1^∙hr^-1^. In the propofol and dexmedetomidine group, infusion rate of propofol was decreased to 25 mg∙kg^-1^∙hr^-1^ and dexmedetomidine was injected 30 μg∙kg^-1^∙hr^-1^ for 10 min (5 μg∙kg^-1^) followed by an infusion of 3.5 μg∙kg^-1^∙hr^-1^. Rectal (core) temperature was maintained at 39 ± 0.5°C by a heating blanket. Arterial blood pressure, heart rate and rectal temperature were recorded using Dynascope DS-7101 L (Fukuda Denshi, Tokyo, Japan).

The animals’ heads were shaved, and a four-electrode pediatric BIS sensor was applied according to the previous method [3, 5]. One electrode was placed 1 cm caudal to the eye socket and others were placed on the head. All electrode impedance levels were kept below 7.5 kΩ. BIS values were obtained from an A-2000 monitor (Version XP-3.12, Aspect Medical Systems, Norwood, MA, USA). The BIS smoothing rate was set at 15 seconds. BIS is a unitless value that ranges from 0 to 100, with values between 40 and 60 indicating a suitable hypnotic depth for surgery in humans [6].

After surgical procedures, 40 minutes were allowed for stabilization. Then measurements of BIS and other parameters such as arterial blood pressure, heart rate and rectal temperature were started. In the sevoflurane group, the inhaled concentration was progressively increased by 0.2% at 10-minute intervals. In propofol and propofol + dexmedetomidine groups, the propofol infusion rate was progressively increased by 5 mg∙kg^-1^∙hr^-1^ at 10-minute intervals. In the sevoflurane + dexmedetomidine group, sevoflurane was initially inhaled at 1.0% and was sequentially increased by 0.5%. BIS and other parameters such as arterial blood pressure, heart rate and rectal temperature were recorded 10 minutes after each dose adjustment. At the end of the experiment, each animal was killed by KCl infusion.

Systolic blood pressures, heart rates, and core temperatures were expressed as means ± standard deviations. Those data were analyzed using analysis of variance followed by a *post hoc* Tukey’s test. The BIS data was assessed using logistic regression, and mean and 95% confidence intervals (CI) were presented. Correlations between each measurement were examined using a scatter graph and linear regression. A *P* value less than 0.05 was considered statistically significant.

## Results

In the sevoflurane group, systolic blood pressure progressively decreased as a function of sevoflurane concentration (Table 1). In the sevoflurane + dexmedetomidine group, systolic blood pressure and heart rate were significantly lower than in the sevoflurane group (Table 1). Blood pressure and heart rate were similarly decreased in the propofol + dexmedetomidine group compared with those in the propofol group (Table 2).

**Table 1:** **Systolic blood pressure, heart rate and body temperature in the sevoflurane and sevoflurane + dexmedetomidine groups**

	Sevoflurane	Sevoflurane + dexmedetomidine	***P***
SBP (mmHg) S 1%	131 ± 24	93 ± 15	0.0036
SBP (mmHg) S 2%	112 ± 14	86 ± 16	0.0068
SBP (mmHg) S 3%	107 ± 19	84 ± 21	0.0343
SBP (mmHg) S 4%	107 ± 14	92 ± 16	NS
SBP (mmHg) S 5%	101 ± 7*	79 ± 12	0.0015
HR (bpm) S 1%	252 ± 43	222 ± 62	NS
HR (bpm) S 2%	258 ± 71	204 ± 36	0.0385
HR (bpm) S 3%	254 ± 59	227 ± 25	NS
HR (bpm) S 4%	239 ± 38	250 ± 32	NS
HR (bpm) S 5%	219 ± 5	267 ± 37	0.0058
BT (°C) S 1%	38.6 ± 0.8	38.5 ± 0.3	NS
BT (°C) S 2%	38.8 ± 1.0	38.6 ± 0.3	NS
BT (°C) S 3%	38.9 ± 1.2	38.7 ± 0.4	NS
BT (°C) S 4%	38.8 ± 0.1	39.0 ± 0.4	NS
BT (°C) S 5%	38.6 ± 0.4	38.5 ± 0.5	NS

SBP = systolic blood pressure, HR = heart rate, bpm = beats per minutes, BT = body temperature, S = sevoflurane concentration, NS not significant, *P <0.05 versus S 1%.

**Table 2:** **Systolic blood pressure, heart rate and body temperature in the propofol and propofol + dexmedetomidine groups**

	Propofol	Propopol + Dexmedetomidine	***P***
SBP (mmHg) P 50	121 ± 14	107 ± 20	NS
SBP (mmHg) P 60	112 ± 13	105 ± 15	NS
SBP (mmHg) P 70	125 ± 11	108 ± 27	0.0473
SBP (mmHg) P 80	123 ± 8	111 ± 13	NS
SBP (mmHg) P 90	116 ± 7	102 ± 17	0.0344
SBP (mmHg) P 100	117 ± 9	95 ± 13	< 0.0001
HR (bpm) P 50	264 ± 29	215 ± 39	0.0064
HR (bpm) P 60	242 ± 34	192 ± 41	0.0019
HR (bpm) P 70	273 ± 19	208 ± 32	< 0.0001
HR (bpm) P 80	279 ± 24	206 ± 20	0.0001
HR (bpm) P 90	280 ± 21	221 ± 18	< 0.0001
HR (bpm) P 100	267 ± 21	218 ± 23	< 0.0001
BT (°C) P 50	39.1 ± 0.4	39.2 ± 0.6	NS
BT (°C) P 60	39.0 ± 0.5	39.1 ± 0.6	NS
BT (°C) P 70	38.9 ± 0.5	39.2 ± 0.6	NS
BT (°C) P 80	39.2 ± 0.4	39.3 ± 0.4	NS
BT (°C) P 90	39.1 ± 0.3	39.2 ± 0.6	NS
BT (°C) P 100	38.6 ± 0.6	39.3 ± 0.7	NS

SBP = systolic blood pressure, HR = heart rate, bpm = beats per minutes, BT = body temperature, P = propofol, 50, 60, 70, 80, 90, 100 = 50, 60, 70, 80, 90, 100 mg∙kg^-1^∙hr^-1^, NS not significant.

**Figure 1:**
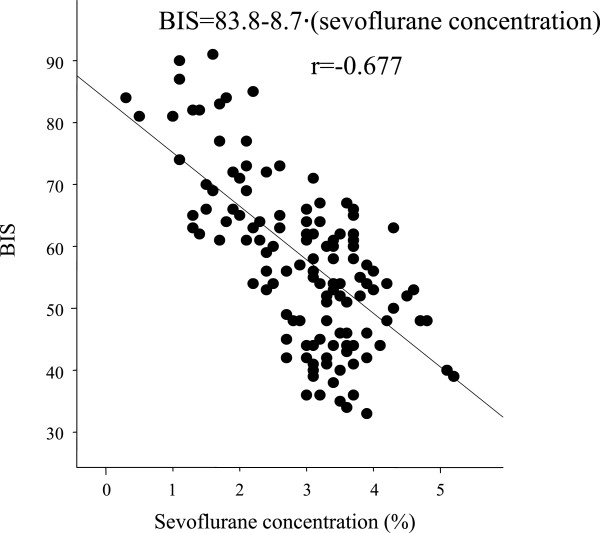
**Linear regression between bispectral index and end-tidal sevoflurane concentration in rabbits anesthetized with sevoflurane: BIS = 84 - 8.7**∙**[sevoflurane (%)], r = -0.68.**

**Figure 2:**
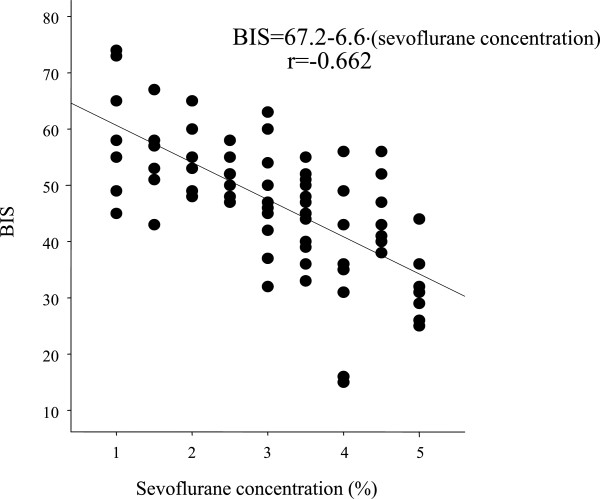
**Linear regression between bispectral index and end-tidal sevoflurane concentration in rabbits anesthetized with sevoflurane and dexmedetomidine.** Line represents linear correlation: BIS = 67.2 - 6.6∙[sevoflurane (%)], r = -0.662.

**Figure 3:**
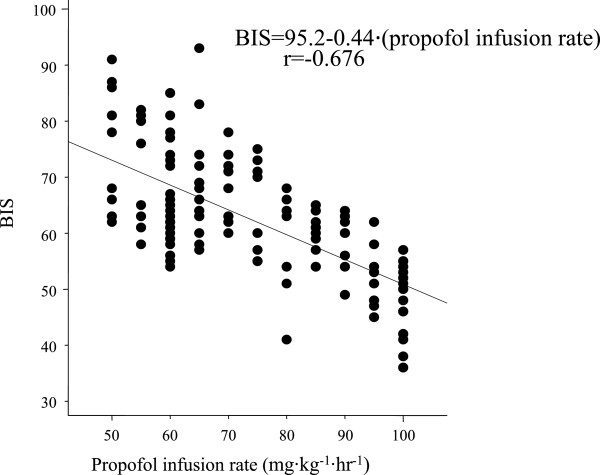
**Linear regression between bispectral index and propofol infusion rate in rabbits anesthetized with propofol: BIS = 95 - 0.44**∙**[Propofol (mg**∙**kg** ^**-1**^∙**hr** ^**-1**^ **)], r = -0.676.**

**Figure 4:**
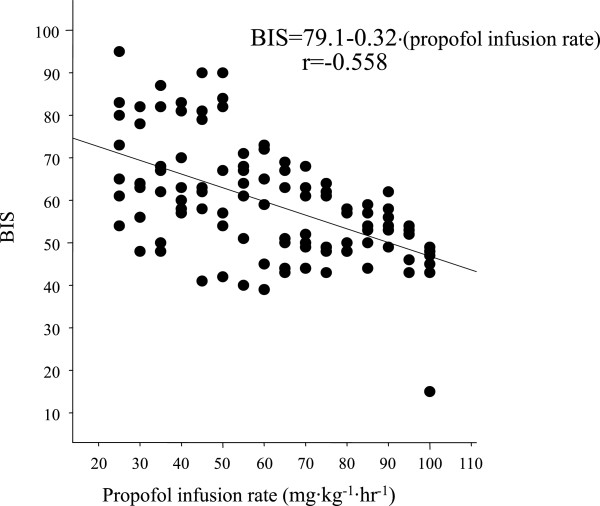
**Linear regression between bispectral index and propofol infusion rate in rabbits anesthetized with propofol and dexmedetomidine: BIS = 79 - 0.32**∙**[Propofol (mg**∙**kg** ^**-1**^∙**hr** ^**-1**^ **)], r = -0.558.**

BIS values correlated with end-tidal sevoflurane concentrations in the sevoflurane group (BIS =83.8 - 8.7∙[sevoflurane (%)], r = -0.677; Figure 1) and the sevoflurane + dexmedetomidine group (BIS =67.2 - 6.6∙[sevoflurane (%)], r = -0.662; Figure 2). The BIS value was also highly correlated with propofol infusion rates in the propofol group (BIS = 95.2 - 0.44∙[Propofol (mg∙kg^-1^∙hr^-1^)], r = -0.676; Figure 3) and the propofol + dexmedetomidine group (BIS = 79.1 - 0.32∙[Propofol (mg∙kg^-1^∙hr^-1^)], r = -0.558; Figure 4).

The sevoflurane concentration required to produce a BIS value of 50 was 3.9 ± 0.2% (95% CI, 3.7-4.1%) in the sevoflurane group (Figure 1) and 2.6 ± 0.3% (95% CI, 2.3-2.9%) in the sevoflurane + dexmedetomidine group (Figure 2). The propofol infusion rate required to produce a BIS value of 50 was 102 ± 5 mg∙kg^-1^∙hr^-1^ (95% CI, 97–107 mg∙kg^-1^∙hr^-1^) in the propofol group (Figure 3) and 90 ± 10 mg∙kg^-1^∙hr^-1^ (95% CI, 80–100 mg∙kg^-1^∙hr^-1^) in the propofol + dexmedetomidine group (Figure 4).

## Discussion

Perhaps unsurprisingly, various species require different anesthetic concentrations. However, the potency of volatile anesthetics differs only slightly across species, with similar concentrations being required for insects, amphibian, rodents, and humans [7–9]. In contrast, requirements for intravenous anesthetics vary considerably. For example, many laboratory animals such as rodents require opioid and ketamine doses an order-of-magnitude greater than human patients [10, 11].

The classical measure of volatile anesthetic potency is the Minimum Alveolar Concentration (MAC) which is the partial pressure (or gas percentage) in the blood which prevents 50% of subjects from moving in response to a super-maximal painful stimulus such as a skin incision. End-tidal concentrations represent alveolar concentration which is in equilibrium with blood and thus brain; end-tidal concentrations thus represent effect-site concentrations. MAC is used to compare the potency of various volatile anesthetics, as well as to evaluate the effects of ancillary drugs [12], mutations [13], and ethnicity [14]. Although MAC is not defined for intravenous anesthetics, it is possible to determine the plasma concentration that prevents movement in 50% of human subjects [15].

While MAC is the oldest and best-established single measure of volatile anesthetic effect, it is hardly the only one. In fact, “anesthesia” is not a unitary function; in addition to lack of movement, it incorporates amnesia, analgesia, and hemodynamic control. Various brain function monitors have thus been developed in an effort to provide a more global measure of anesthetic (or at least hypnotic) effect. Spectral edge frequency 95 (SEF95) was the first generally accepted electroencephalographic measure [16], but the BIS is now by far the most commonly used measure of hypnotic effect [17]. And unlike many other measures, it appears to be generally reliable across a variety of anesthetic drug classes and combinations of anesthetic drugs including analgesics including propofol, isoflurane, thiopental, or midazolam, supplemented with various amounts for opioids and nitrous oxide [18].

In the previous human studies, BIS values were linearly correlated with effect-site sevoflurane concentration [19], and propofol blood concentration [20]. We found that BIS values in rabbits were also linearly related to end-tidal sevoflurane concentration and the propofol dose. The correlation line slopes of sevoflurane and propofol were similar to those observed in previous human studies [19, 20]. Comparable slopes suggest that BIS values are a reasonable gauge of sedation depth in rabbits anesthetized with sevoflurane or propofol.

The MAC of sevoflurane in rabbits is between 3.7 and 4.1% [21, 22]. Interestingly, the end-tidal sevoflurane concentrations that produced BIS = 50 was almost identical at 3.9%. This result is consistent with the general finding that volatile anesthetic potency is comparable across a wide variety of species.

The hypnotic dose of propofol in rabbits remains controversial. Luo et al [23] used 24–48 mg∙kg^-1^∙hr^-1^ of propofol with ketamine and fentanyl. Baumgartner et al [24] used 90–102 mg∙kg^-1^∙hr^-1^ of propofol with fentanyl or dipyrone. Martín-Cancho et al [3] reported that propofol dose of 36 mg∙kg^-1^∙hr^-1^ was associated with BIS values of 69.1. From our results, dose of propofol that makes BIS at 70 is calculated to be 57.3 mg∙kg^-1^∙hr^-1^ (Figure 3). This dose is 1.6 times higher than that in the previous study, however, the dose is not extremely high. Our results may concur with the previous study. Results of previous studies suggest that the necessary infusion rate for propofol in rabbits varies from 24 to 100 mg∙kg^-1^∙hr^-1^[23, 24]. We found that infusion rate of propofol that adjusted BIS value at 50 was 101 mg∙kg^-1^∙hr^-1^, suggesting that the higher published doses may be most suitable. We note, though, that dose must be interpreted in the context of ancillary medications which themselves contribute to anesthetic effect and thus influence BIS values. In patients, a more typical dose would be 5.3 mg∙kg^-1^∙hr^-1^[25]. Rabbits thus require more propofol than humans.

Dexmedetomidine decreases both the MAC of sevoflurane and propofol requirements in humans [26, 27]. For example, dexmedetomidine decreases BIS in swine given a constant infusion of propofol [28]. Dexmedetomidine similarly decreases the MAC of sevoflurane by about 50% in ponies [29]. Our results in rabbits are generally consistent, with dexmedetomidine reducing sevoflurane requirement by 33% and propofol requirement by 14%.

Continuous infusion rate of dexmedetomidine at 0.25-1 μg∙kg^-1^∙hr^-1^ is recommended for humans. Kilic et al [30] reported that the dose of dexmedetomidine was 10 μg ∙kg^-1^ followed by a maintenance dose of 10 μg∙kg^-1^∙hr^-1^. Chang et al [31] reported that infusion dose of dexmedetomidine was at 2 μg∙kg^-1^∙hr^-1^. Rabbits may need large amount of dexmedetomidine compared with human. Used dose of dexmedetomidine at 3.5 μg∙kg^-1^∙hr^-1^ in this study could be adaptable for rabbits.

Blood pressure and heart rate were essentially unchanged by sevoflurane and propofol alone. Even at 100 mg∙kg^-1^ of propofol did not induce hypotension. In contrast, dexmedetomidine decreased blood pressure and heart rate just as would be expected from a central alpha-2 receptor agonist that also acts on pre-synaptic alpha-2 adrenergic receptor and suppresses norepinephrine release [32, 33]. The employed dose of dexmedetomidine at 3.5 μg∙kg^-1^∙hr^-1^ could be the origin of decrease in blood pressure. However, although propofol 100 mg∙kg^-1^∙hr^-1^ combined with dexmedetomidine 3.5 mcg∙kg^-1^∙hr^-1^ decreased blood pressure, that combination did not induce severe hypotension. In contrast to our steady-state measurements, initiating dexmedetomidine — or rapid dose increases — can increase blood pressure in response to unopposed stimulation of peripheral alpha-2 receptors. However, the increase is usually transient and of minimal clinical consequence [34].

In animal studies, airway management is crucial. Su et al [35] reported that tracheal intubation in rabbits was difficult because of their features such as limited mandibular range, a large tongue and prominent incisors. We also experienced that tracheal intubation in rabbits was very difficult. Tracheotomy is commonly used method for airway management for rabbit studies, and it is a reliable method for protecting the airway. Therefore, we employed tracheotomy.

A limitation of our study is that it depends critically on BIS being valid in rabbits, and a BIS value of 50 representing optimal anesthetic depth. Although not extensively validited, BIS appears to work well in animals [4, 5] including rabbits [3]. We also assumed that BIS values have comparable meanings with sevoflurane, propofol, and dexmedetomodine. BIS is not a “universal tool”. For example, nitrous oxide [36] and ketamine [37] have little effects on BIS, although they are perfectly good anesthetics. However, nitrous oxide and ketamine appear to be exceptions, with most other anesthetics having roughly comparable effects on BIS.

The rabbits in our study had a tracheostomy, but did not experience the kind of pain that results from major surgery. During major surgery, either sevoflurane or propofol would need to be accompanied by an analgesic, usually an opioid [38, 39]. And finally, we report propofol dose rather than plasma concentration. Concentration would provide a better pharmacokinetic estimate, and in humans effect-site concentration would be calculated from established models. But such models do not exist for rabbits, and dose is more clinically useful than blood concentration which cannot be measured in real time.

## Conclusions

The sevoflurane concentration at BIS = 50 was 3.9% in the sevoflurane group and 2.6% in the sevoflurane + dexmedetomidine group. The propofol infusion rate to make BIS = 50 was 101 mg∙kg^-1^∙hr^-1^ in the propofol group, and 90 mg∙kg^-1^∙hr^-1^ in the propofol + dexmedetomidine group. The optimal end-tidal concentration of sevoflurane alone was thus 3.9%, and optimal infusion rate for propofol alone was 101 mg∙kg^-1^∙hr^-1^. Dexmedetomidine reduced sevoflurane requirement by 33% and propofol requirement by 11%.

## Authors’ information

Yoshihide Terada: Instructor, Department of Anesthesiology, Faculty of Medicine, University of Yamanashi.

Tadahiko Ishiyama: Associate Professor and Chair, Surgical Center, University of Yamanashi Hospital.

Nobumasa Asano: Instructor, Department of Anesthesiology, Faculty of Medicine, University of Yamanashi.

Masakazu Kotoda: Instructor, Surgical Center, University of Yamanashi Hospital.

Kodai Ikemoto: Instructor, Department of Anesthesiology, Faculty of Medicine, University of Yamanashi.

Noriyuki Shintani: Instructor, Department of Anesthesiology, Faculty of Medicine, University of Yamanashi.

Daniel I Sessler; Michael Cudahy Professor and Chair, Department of Outcomes Research, The Cleveland Clinic.

Takashi Matsukawa: Professor and Chair, Department of Anesthesiology, Faculty of Medicine, University of Yamanashi.

## Abbreviations

BIS: Bispectral index
CI: Confidence intervals
MAC: Minimum Alveolar Concentration
SEF95: Spectral edge frequency 95.
